# Impact of forest plantation on methane emissions from tropical peatland

**DOI:** 10.1111/gcb.15019

**Published:** 2020-02-20

**Authors:** Chandra S. Deshmukh, Dony Julius, Chris D. Evans, Nardi, Ari P. Susanto, Susan E. Page, Vincent Gauci, Ari Laurén, Supiandi Sabiham, Fahmuddin Agus, Adibtya Asyhari, Sofyan Kurnianto, Yogi Suardiwerianto, Ankur R. Desai

**Affiliations:** ^1^ Asia Pacific Resources International Ltd. Kabupaten Pelalawan Indonesia; ^2^ Centre for Ecology and Hydrology Bangor UK; ^3^ Centre for Landscape and Climate Research School of Geography, Geology and the Environment University of Leicester Leicester UK; ^4^ Birmingham Institute of Forest Research (BIFoR) School of Geography, Earth and Environmental Sciences University of Birmingham Birmingham UK; ^5^ School of Forest Sciences Faculty of Science and Forestry University of Eastern Finland Joensuu Finland; ^6^ Department of Soil Science and Land Resource Institut Pertanian Bogor Bogor Indonesia; ^7^ Indonesian Center for Agricultural Land Resources Research and Development Bogor Indonesia; ^8^ Department of Atmospheric and Oceanic Sciences University of Wisconsin‐Madison Madison WI USA

**Keywords:** *Acacia crassicarpa*, eddy covariance measurements, forest plantation, Indonesia, land‐use change, methane emissions, peatland management, tropical peatlands

## Abstract

Tropical peatlands are a known source of methane (CH_4_) to the atmosphere, but their contribution to atmospheric CH_4_ is poorly constrained. Since the 1980s, extensive areas of the peatlands in Southeast Asia have experienced land‐cover change to smallholder agriculture and forest plantations. This land‐cover change generally involves lowering of groundwater level (GWL), as well as modification of vegetation type, both of which potentially influence CH_4_ emissions. We measured CH_4_ exchanges at the landscape scale using eddy covariance towers over two land‐cover types in tropical peatland in Sumatra, Indonesia: (a) a natural forest and (b) an *Acacia crassicarpa* plantation. Annual CH_4_ exchanges over the natural forest (9.1 ± 0.9 g CH_4_ m^−2^ year^−1^) were around twice as high as those of the *Acacia* plantation (4.7 ± 1.5 g CH_4_ m^−2^ year^−1^). Results highlight that tropical peatlands are significant CH_4_ sources, and probably have a greater impact on global atmospheric CH_4_ concentrations than previously thought. Observations showed a clear diurnal variation in CH_4_ exchange over the natural forest where the GWL was higher than 40 cm below the ground surface. The diurnal variation in CH_4_ exchanges was strongly correlated with associated changes in the canopy conductance to water vapor, photosynthetic photon flux density, vapor pressure deficit, and air temperature. The absence of a comparable diurnal pattern in CH_4_ exchange over the *Acacia* plantation may be the result of the GWL being consistently below the root zone. Our results, which are among the first eddy covariance CH_4_ exchange data reported for any tropical peatland, should help to reduce the uncertainty in the estimation of CH_4_ emissions from a globally important ecosystem, provide a more complete estimate of the impact of land‐cover change on tropical peat, and develop science‐based peatland management practices that help to minimize greenhouse gas emissions.

## Graphical abstract

We measured methane exchanges at the landscape scale using eddy covariance towers over a natural forest and an *Acacia crassicarpa* plantation in tropical peatland in Sumatra, Indonesia. Groundwater level (GWL) controls diurnal and seasonal variability in methane emissions. Annual methane emissions over the natural forest were around twice as high as those of the *Acacia* plantation. Lower methane emissions over the *Acacia* plantation may be attributed to lower GWL. Results highlight that tropical peatlands are significant methane sources, and probably have a greater impact on global atmospheric methane concentrations than previously thought.

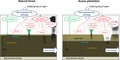

## 1 INTRODUCTION

Methane (CH_4_) is the second most important anthropogenic greenhouse gas after carbon dioxide (CO_2_) and its concentration is continuing to increase (Dlugokencky, 2019; Nisbet et al., 2019). The global warming potential (GWP) of CH_4_ is 34 times that of CO_2_ on a 100 year basis when including climate–carbon feedbacks (Myhre et al., 2013). Due to its short atmospheric life span of about 10 years and relatively high GWP, there is increasing interest in reducing CH_4_ emissions in order to meet global temperature targets (Collins et al., 2018). Current and future regional and global CH_4_ budgets and mitigation strategies require better quantitative and process‐based understanding of CH_4_ sources, pathways, and removals under climate and land‐use change (Saunois et al., 2016).

Natural wetlands are the single largest source of atmospheric CH_4_ (Kirschke et al., 2013; Poulter et al., 2017; Saunois et al., 2016). The coastal lowlands of Southeast Asia host one‐third of tropical peatlands, with the majority located in Indonesia (Gumbricht et al., 2017; Page, Rieley, & Banks, 2011), and they represent unrecognized and poorly understood components of the CH_4_ cycle (Pangala, Moore, Hornibrook, & Gauci, 2013; Wong et al., 2018). Since the 1980s, extensive areas of Southeast Asian peatlands have experienced land‐cover changes (Miettinen, Shi, & Liew, 2016; Wijedasa et al., 2018), driven by transmigration, local population growth, and ongoing economic development. The 2015 land‐cover distribution for the insular Southeast Asian peatlands reveals that half of all former peatland forest is managed as either small‐holder agriculture or industrial plantation, while around 29% is characterized as intact or degraded natural peat swamp forest (Miettinen et al., 2016). The remaining 21% of the peatlands are covered by open undeveloped areas, fern, low/tall shrub, and secondary regrowth forest (Miettinen et al., 2016). Agriculture and forest plantation on peatlands require the maintenance of groundwater level (GWL) below the root zone to support the required level of productive growth. Maintaining the GWL below the surface alters the CH_4_ dynamic by weakening the potential for CH_4_ production and increasing the potential for CH_4_ oxidation in the upper peat layers (Furukawa, Inubushi, Ali, Itang, & Tsuruta, 2005; Melling, Hatano, & Goh, 2005). Given the potential importance of tropical peatlands in global CH_4_ budgets, it is important to understand any effects of land‐cover changes on CH_4_ emissions from tropical peatlands.

When the balance between CH_4_ production and consumption is positive, CH_4_ can be emitted to the atmosphere via: (a) diffusion from soil and water surfaces, (b) ebullition from water surfaces, or (c) vegetation‐mediated transport through aerenchymatous and air‐filled tissues in herbaceous plants and trees (Jauhiainen & Silvennoinen, 2012; Pangala et al., 2013). In addition, CH_4_ can be emitted from terrestrial arthropods such as termites (Jeeva, Bignell, Eggleton, & Maryati, 1999) and plants producing CH_4_ in aerobic conditions (Keppler, Hamilton, Brass, & Röckmann, 2006). The contribution of each pathway to total ecosystem CH_4_ exchange varies within and among peatland ecosystems depending on surface microtopography (hummock vs. hollow), GWL, peat temperature, vegetation composition and structure, and land‐use practices (Melling et al., 2005; Pangala et al., 2013). Variation in plant physiological processes driven by solar radiation might substantially influence vegetation‐mediated transports as observed in northern peatlands (Kim, Verma, Billesbach, & Clement, 1998; Long, Flanagan, & Cai, 2010; Nisbet et al., 2009; van der Nat, Middelburg, van Meteren, & Wielemakers, 1998). Thus, significant spatial and temporal variability in CH_4_ emissions from tropical peatlands can be anticipated, yet available data rarely allow analysis of how such variability influences annual emissions.

Measurements of CH_4_ emissions from tropical peatlands are sparse and have focused mainly on soil surfaces based on small‐scale chamber measurements (Furukawa et al., 2005; Hadi et al., 2005; Inubushi, Hadi, Okazaki, & Yonebayashi, 1998; Ishikura et al., 2019; Jauhiainen, Limin, Silvennoinen, & Vasander, 2008; Melling et al., 2005). The large and heterogeneous nature of forest vegetation, together with dynamic biotic and abiotic processes, makes it difficult to measure vegetation‐mediated transport accurately using chamber systems (Barba et al., 2018). Notably, vegetation‐mediated transport, principally through trees, could be 62%–87% of total ecosystem CH_4_ exchange (Pangala et al., 2013) and represents a “science frontier” in our understanding of biosphere–atmosphere exchange in forests (Barba et al., 2018).

Knowledge of the magnitude of CH_4_ exchanges including all existing sources and removals in tropical peatland ecosystems is limited (Pangala et al., 2013; Sakabe, Itoh, Hirano, & Kusin, 2018; Tang et al., 2018; Wong et al., 2018). Many process‐based CH_4_ models lack sufficient details in their treatment and parameterization of transport pathways to derive reliable emissions estimates (Gedney, Huntingford, Comyn‐Platt, & Wiltshire, 2019; Parker et al., 2018). This leads to uncertainty in estimates of the current and future contribution of tropical peatlands to regional and global CH_4_ budgets (Saunois et al., 2016).

Given these uncertainties, we need to improve our understanding of the spatiotemporal and environmental variability that drive exchange strength and direction in order to better understand the potential CH_4_ exchanges that may result from any future climate or land‐use change scenarios. Micrometeorological methods (such as eddy covariance) provide half‐hourly measurements of turbulent CH_4_ exchanges between an entire ecosystem and the atmosphere above the vegetation canopy (Aubinet et al., 2000). Hence, eddy covariance measurements incorporate all existing CH_4_ sources and removals that can vary significantly within an ecosystem in both space and time arising from variation in environmental conditions.

In the above context, we used the eddy covariance technique to measure net ecosystem CH_4_ exchange over two land‐covers in a single peatland hydrological unit on the Kampar Peninsula in Sumatra, Indonesia: (a) a natural forest, and (b) a forest plantation (*Acacia crassicarpa*). Measurements were conducted for more than four site‐years (October 2016–May 2019 over the *Acacia* plantation and June 2017–May 2019 over the natural forest). The main objectives of this study were to: (a) determine the magnitudes of CH_4_ exchanges from tropical peatlands while incorporating all existing sources and removals, and (b) understand the link between temporally varying CH_4_ exchanges and associated changes in the environmental controls.

We hypothesized that a lower GWL would reduce vegetation‐mediated CH_4_ transport to the atmosphere in the managed peatland. We evaluated this hypothesis over timescales ranging from diurnal to annual. These results were then used to quantify the impact of *Acacia* plantation, considering the change in CH_4_ exchanges due to the associated altered landscape, as one component of the ecosystem greenhouse gas balance. Finally, we considered the relevance of these results for tropical peatland greenhouse gas emissions reporting, climate change mitigation policies and land‐use management.

## 2 MATERIALS AND METHODS

### 2.1 Study area

**Figure 1:**
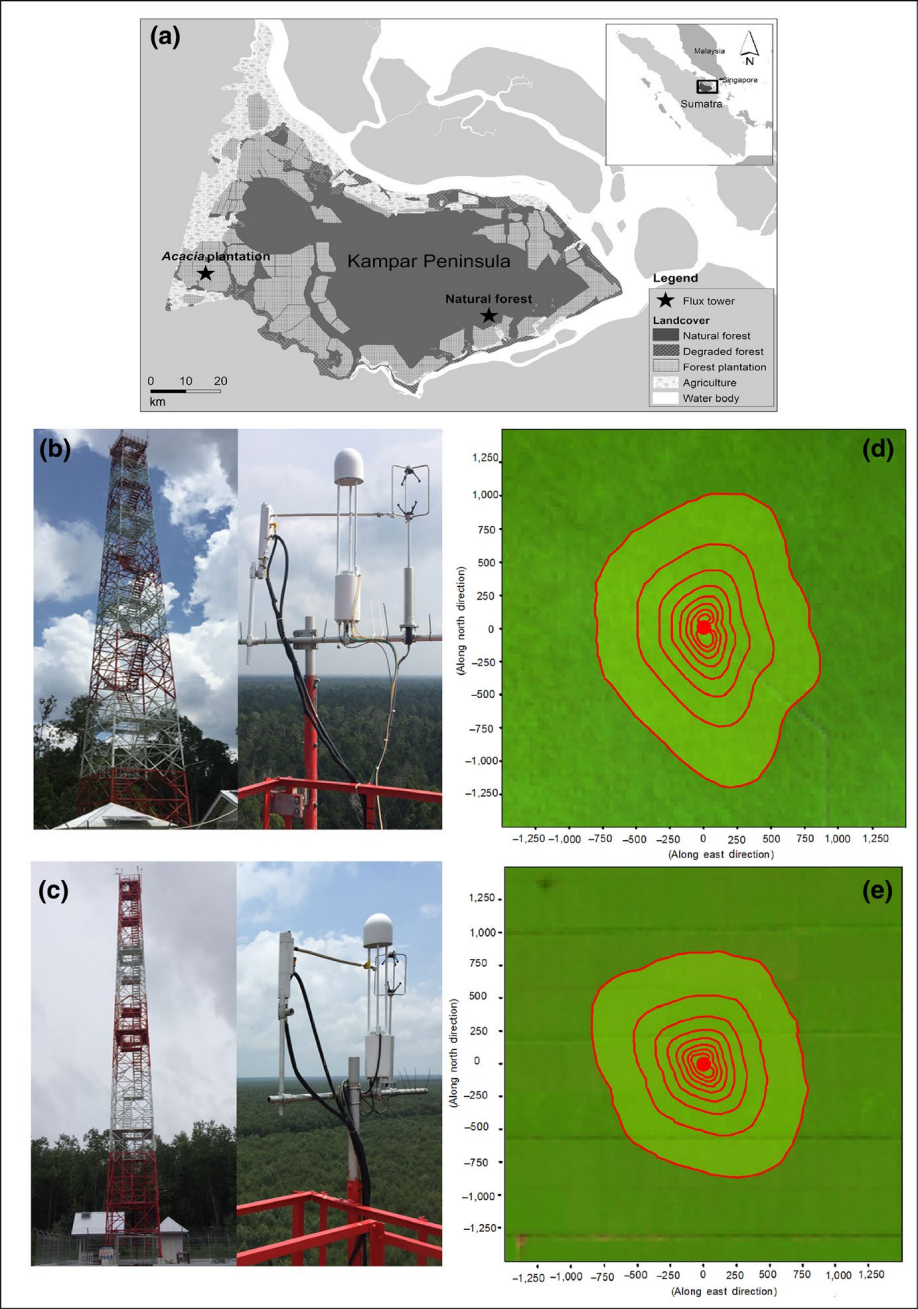
Land‐cover map of the Kampar Peninsula, Sumatra, Indonesia and the location of research flux tower sites (a), photos of the eddy covariance instruments installed at the top of the tower at the natural forest (b), and the *Acacia* plantation (c), and integrated eddy covariance footprint contour lines from 10% to 80% in 10% intervals over the natural forest for June 2017–May 2019 (d), and the *Acacia* plantation for October 2016–May 2019 (e)

The Kampar Peninsula is a coastal tropical peatland of around 700,000 ha (Figure 1a). This ombrotrophic (acidic and nutrient‐poor) peatland is largely formed within the past 8,000 years (Dommain, Couwenberg, & Joosten, 2011). The study area has a humid tropical climate (warm year‐round) with average monthly air temperature ranging from 29 to 32°C (Badan Meteorologi, Klimatologi dan Geofisika, 1994–2017 data). Average annual rainfall for the last 5 years (2014–2018) is ~1,800 mm with two wet seasons (March–April and October–December) and two dry seasons (January–March and May–August). The peninsula is characterized by a large, relatively intact central forest area surrounded by a mosaic of smallholder agricultural land (largely oil palm, *Elaeis guineensis*), and industrial fiber wood plantation (largely *A. crassicarpa*), smaller secondary forest areas, and undeveloped open and degraded land (Figure 1a; Miettinen et al., 2016). Natural forest and *Acacia* plantation together occupy around 80% of the peninsula (Figure 1a).

Above‐canopy eddy covariance flux towers were established at the *Acacia* plantation and the natural forest in 2016 and 2017, respectively, for the purpose of measuring net ecosystem CO_2_ and CH_4_ exchange (Figure 1b,c; note that CO_2_ flux measurements will be reported separately). The terrain around the towers is flat (slope <0.05%) and land‐cover and topography are homogenous for at least 3 km in all directions at both sites, ensuring a good fetch and a consistent land‐cover–related signal regardless of wind direction. The relatively close proximity of the natural forest and the *Acacia* plantation sites (~80 km apart) within the same peatland hydrological unit avoids potentially confounding variables such as climatic differences, past natural succession, and to some extent geomorphological formation (Figure 1a). Thus, although it is inherently difficult and expensive to replicate flux measurements using the eddy covariance technique, our sites should provide a robust and unbiased basis for evaluating the impact of land‐cover change (from peat swamp forest to *Acacia* plantation) on CH_4_ exchanges.

The natural forest is characterized as pristine peat swamp forest (Miettinen et al., 2016). The forest structure is mixed, and the canopy is uneven with the tallest canopy in a range of 28–35 m. Tree density with diameter at breast height >5 cm was 1,343 trees per hectare. The dominant tree species of the overstory are *Shorea uliginosa*, *Calophyllum ferrugineum*, and *Syzygium* spp.; together they represent around 75% of the overstory vegetation (Table 1). The understory is dominated by *Pandanus* spp., *Cyrtostachys renda*, and *Nepenthes* spp. The forest floor is uneven with a hummock‐hollow microtopography, and covered with tree debris, root mat, and leaf litter. Hollow surfaces are often 20–40 cm lower than hummock tops. The average area ratio of hollow to hummock was 3:1 around the tower. The surface peat type is fibric and the average peat thickness is ~9 ± 1 m in the area surrounding the tower. The surface peat pH is 3.6 ± 0.1 and the GWL fluctuates seasonally with the rainfall variation (see Section 3). An integrated climatologic footprint analysis (Kljun, Calanca, Rotach, & Schmid, 2015) indicated that approximately 80% of fluxes were derived within 1,200 m in the upwind direction (Figure 1d), and thus originated within the pristine peat swamp forest as characterized by Miettinen et al. (2016).

**Table 1:** Site characteristics. Value represents average ± standard deviation

Parameter	Natural forest	*Acacia* plantation
Tower location	Latitude: 0°23′42.735″N Longitude: 102°45′52.382″E	Latitude: 0°30′57.221″N Longitude: 102°2′11.090″E
Tower height (m)	48	40
Average canopy height (m)	32 ± 6	17 ± 6
Dominant understory species	*Nepenthes, Pandanus, Cyrtostachys renda*	Not available
Dominant trees species	*Shorea uliginosa*, *Calophyllum ferrugineum*, *Syzygium* spp., *Camnosperma macrophylla*, *Tetramerista glabra*, *Palaquium burckii*	*Acacia crassicarpa*
Peat depth (m)	9 ± 1.0	7 ± 0.8
Surface (0–50 cm) peat type	Fibric	Hemic
Surface (0–50 cm) peat bulk density (g/cm^3^)	0.08 ± 0.03	0.14 ± 0.02
Surface (0–50 cm) peat pH	3.6 ± 0.10	3.4 ± 0.03

At the forest plantation, *A. crassicarpa* trees are grown for fiber production on a 5 year rotation from planting to harvesting. When measurements began in October 2016, the trees were already at the end of the plantation cycle. In March–April 2017, the mature trees, which had achieved an average height of 20 m, were harvested. Replanting at a density of 1,667 trees per hectare (3 m × 2 m spacing) took place within 2 weeks after harvesting. One kg boiler wood ash per tree was applied around the seedlings during planting as per the standard operational procedure, without additional fertilizers. In May 2019, 2 years after replanting, the canopy height was ~17 m. The ground surface within the plantation area is relatively even, without a hummock‐hollow microtopography, and with very little understory vegetation. The surface peat type is hemic and the average peat thickness is ~7 ± 0.8 m in the surrounding area of the tower. The surface peat pH is 3.4 ± 0.1. GWLs in plantation are actively managed to support the required level of productive growth via an extensive network of topographically defined water management zones, controlled by outlet sluices, and supported by large‐scale and continuous rainfall and water level monitoring (Evans et al., 2019). Water management zones comprise of ditches and canals (also used for transportation). The integrated climatologic footprint analysis (Kljun et al., 2015) indicated that (a) approximately 80% of fluxes were estimated to occur within 1,000 m in the upwind direction, and thus originated within the *Acacia* plantation; and (b) the water surface of ditches and canals represented 2.1% of the flux footprint (Figure 1e).

### 2.2 Net ecosystem CH_4_ exchange and environmental variables measurements

The eddy covariance system consisted of an open path gas analyzer (LI‐7700, LI‐COR Inc.) to measure the atmospheric CH_4_ concentration, and a three‐dimensional sonic anemometer (WindMaster Pro 3‐Axis Anemometer, Gill Instruments Limited) to measure orthogonal components of wind speed fluctuations. The mirrors of the CH_4_ analyzer were self‐cleaned either at 5:00 (local time) every day or if the received signal strength indicator (RSSI) dropped below 20%. Furthermore, the upper and lower mirrors of the CH_4_ analyzer were manually cleaned on a biweekly basis. Dew condensation, rain, and dirty window events were excluded using an RSSI value of 20% because CH_4_ data become noisy below this threshold (Chu et al., 2014; McDermitt et al., 2011). Water vapor densities were measured using an enclosed path CO_2_/H_2_O analyzer (LI‐7210, LI‐COR Inc.). The sensors were mounted at the top of the tower to ensure complete exposure in all directions (Figure 1b,c). The raw turbulence eddy covariance data were recorded at 10 Hz using an analyzer interface unit (LI‐7550, LI‐COR Inc.) and stored on a removable flash disk (APRO, Industrial Grade USB Flash Disk).

Quantum sensors (LI‐190SL‐50, LI‐COR Inc.) were mounted at the top of the towers to measure the incoming photosynthetic photon flux density (PPFD, µmol m^−2^ day^−1^). Relative humidity (RH, %), and thereby the vapor pressure deficit (VPD, hPa) and air temperature (*T*_air_, °C) were measured using the air temperature and humidity probes (Vaisala HMP155 Humidity Temperature Probe, Vaisala, Inc), which were installed inside a ventilated radiation shield at the top of the towers. Estimates of canopy conductance to water vapor, an indicator of transpiration, were made from measurements of latent heat flux following the approach described in Long et al. (2010).

Daily rainfall (mm/day) rates were measured using three and six manual bucket systems within 10 km distance from the tower location in the natural forest and the *Acacia* plantation, respectively. Manual bucket systems were installed 1.5 m above the ground, in an open area so that rainfall was not intercepted by the tree canopy. Soil temperature (*T*_soil_, °C) was measured at 15 cm below the peat hollow surface using temperature probe (Stevens Hydra Probe II, Stevens Water Monitoring Systems, Inc.) with three replicates at each tower site. GWLs (m) were monitored as the water elevation relative to the ground surface (taking the base of the hollows as a datum) every 30 min using a GWL logger (Solinst Levelogger Model 3001). The GWL logger was placed in a perforated polyvinyl chloride (PVC) tube that was inserted vertically into the peat at a distance approximately 30 m away from the towers. The GWL logger also recorded temperature at 150 cm below the peat surface that is below GWL. Additionally, PVC poles were randomly distributed within a 3 km radius around the tower locations to monitor GWL fortnightly). All meteorological sensors took measurements every second and were recorded as one minute averages with a datalogger (Sutron Model 9210 XLITE, Sutron Corporation).

All measuring systems were powered using solar panels along with a rechargeable battery system (65 Watt Solar Package, SunWize Power & Battery). Owing to the large power requirement and cost of a separate CH_4_ analyzer, we could not conduct CH_4_ profile measurements to calculate CH_4_ storage below the flux measurement height (Finnigan, 2006). In theory, accumulated CH_4_ below the canopy during nighttime is likely to be released and measured by the EC system following the onset of turbulence after sunrise and the bias on annual sums should be negligible (Xu et al., 2019).

### 2.3 Eddy covariance data processing

Net ecosystem CH_4_ exchange (NEE‐CH_4_) was computed from the 10 Hz vertical wind velocity and CH_4_ concentration data using EddyPro software (version 6.2.0, LI‐COR Inc.) at a standard averaging interval of half hour period (Aubinet et al., 2000). A de‐spiking procedure was applied to detect and eliminate individual out‐of‐range values for vertical wind velocity and CH_4_ concentrations (Vickers & Mahrt, 1997). De‐trending was carried out using the block averaging method. A coordinate correction was applied to force the average vertical wind velocity to zero by the planar fit method (Wilczak, Oncley, & Stage, 2001). Frequency response loss corrections were applied to compensate the flux losses at different frequencies (Massman, 2000, 2001; Moncrieff, Clement, Finnigan, & Meyers, 2004). Fluctuations in CH_4_ density due to temperature (thermal expansion) and water vapor (dilution) were corrected using the Webb–Pearman–Leuning correction (Webb, Pearman, & Leuning, 1980) and spectroscopic effects taken into account by EddyPro (Burba, Anderson, & Komissarov, 2019). Differences between deployment‐specific variables, that is, sensor separation distance and instrument placement, were considered while processing the data. We adopted the standard meteorological notation whereby a positive value of NEE‐CH_4_ represents a net CH_4_ flux to the atmosphere, and a negative value indicates net CH_4_ uptake from the atmosphere (Aubinet et al., 2000). All NEE‐CH_4_ values in the paper are reported in mass of CH_4_ per unit area per time.

After a set of quality controls, the numbers of high‐quality measurements during the course of the study were 38% and 29% for the natural forest and the *Acacia* plantation, respectively, including measuring system malfunctions due to lightning strikes and power supply failure (Table 2). In other words, we obtained a total of 13,637 and 13,548 half‐hourly measurements that met all quality criteria for the natural forest and the *Acacia* plantation, respectively. We gap‐filled both low‐quality and missing data due to instrument malfunction, as is commonly done in eddy covariance studies.

**Table 2:** Summary of the percentage of half‐hourly net ecosystem CH_4_ exchange data that were removed using various quality control criteria and accepted high quality data

	Natural forest	*Acacia* plantation
Stationarity criteria (Mauder et al., 2013)	19%	24%
Extreme outlier (Papale et al., 2006)	6%	10%
u* threshold (Wutzler et al., 2018)	12%	7%
Instrument malfunction	25%	30%
Accepted high quality data	38%	29%

We applied two gap‐filling approaches (a) mean diurnal course (MDC; Dengel, Levy, Grace, Jones, & Skiba, 2011; Sakabe et al., 2018; Wong et al., 2018) and (b) marginal distribution sampling (MDS; Alberto et al., 2014; Dalmagro et al., 2019; Tang et al., 2018) using the REddyProc package (Wutzler et al., 2018). The MDC is a simple interpolation technique where the missing value is replaced with the averaged value of the adjacent days at exactly that time of day (Falge et al., 2001). The MDS considers the covariation of the fluxes with the environmental variables and the temporal autocorrelation of fluxes. We performed MDS gap filling separately for the daytime (06:00–16:00 hr) and the nighttime (18:00–06:00 hr) data. GWL and PPFD were used during the daytime, whereas GWL and *T*_soil_ above the GWL were used during the nighttime gap‐filling. The emissions were similar from both methods at the natural forest (Mann–Whitney test; *p* = .34, Table 4), whereas the emissions were different from both methods at the *Acacia* plantation (Mann–Whitney test; *p* < .05, Table 4). To provide a conservative estimate, we used the average of the MDC and MDS approaches*.* Flux random uncertainty (*σ*_1_) was calculated following Finkelstein and Sims (2001). The standard deviation of three different flux values derived from friction velocity (u*) thresholds of 5th, 50th, and 95th percentiles were applied as an uncertainty due to u* threshold (*σ*_2_) using the REddyProc package (Wutzler et al., 2018). The flux uncertainty due to gap‐filling (*σ*_3_) was calculated as the standard deviation of the binned records used to fill the missing value (Wutzler et al., 2018). The total uncertainty in NEE‐CH_4_ was calculated with the law of propagation of errors (Deventer et al., 2019). Only high quality measurements were used in the qualitative analysis (Figures 4a,b, 5, and 6) and gap‐filled data were used in the quantitative analysis (Figures 3g,h, 4c,d, and 7 and Table 4).

### 2.4 Statistical analyses

Differences between groups of data were examined using *t* test in GraphPad Prism (GraphPad Software, Inc., v5.04). The choice of the non‐parametric test (Mann–Whitney test compare median values) was dependent on non‐normal behavior of the datasets. All statistical tests used a significance level of 5%.

## 3 RESULTS

### 3.1 Environmental conditions

**Figure 2:**
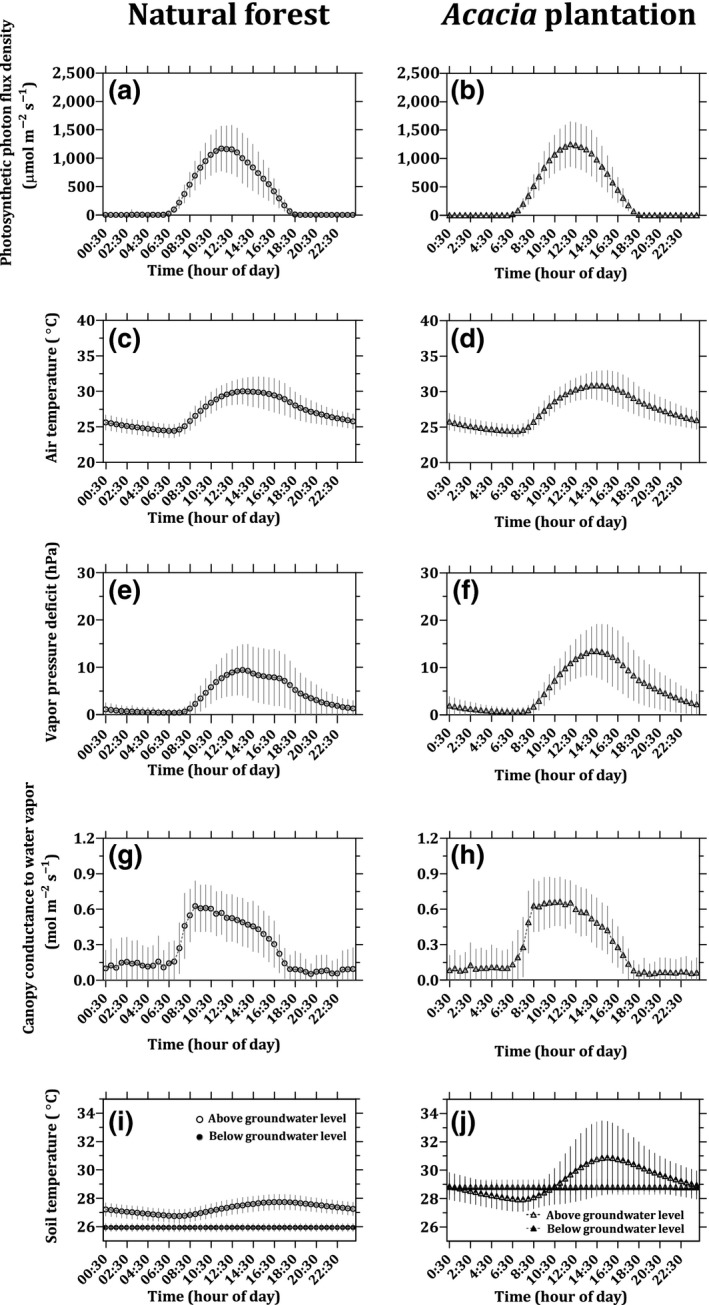
Average diurnal variation in the photosynthetic photon flux density (a, b), air temperature (c, d), vapor pressure deficit (e, f), canopy conductance to water vapor (g, h), and soil temperature (i, j) at the natural forest (left panels) and the *Acacia* plantation (right panels). Data were binned by time of day and then presented for all days during the measurement periods. The error bars show the standard deviation

During the course of the study, the PPFD, *T*_air_, VPD, and canopy conductance to water vapor above the canopy showed typical diurnal patterns reaching their maximum around noon (Figure 2a–h). No significant diurnal variation in *T*_soil_ below the GWL was observed at either site (Figure 2i,j). The diurnal variation in *T*_soil_ above the GWL was small (<1°C) at the natural forest, due to the closed canopy and high GWL (Figure 2i). Before canopy closure, the *Acacia* plantation showed a clear diurnal variation (amplitude of 3°C) in the *T*_soil_ above the GWL, but after canopy closure, the observed diurnal *T*_soil_ above the GWL amplitude was similar to the natural forest.

**Figure 3:**
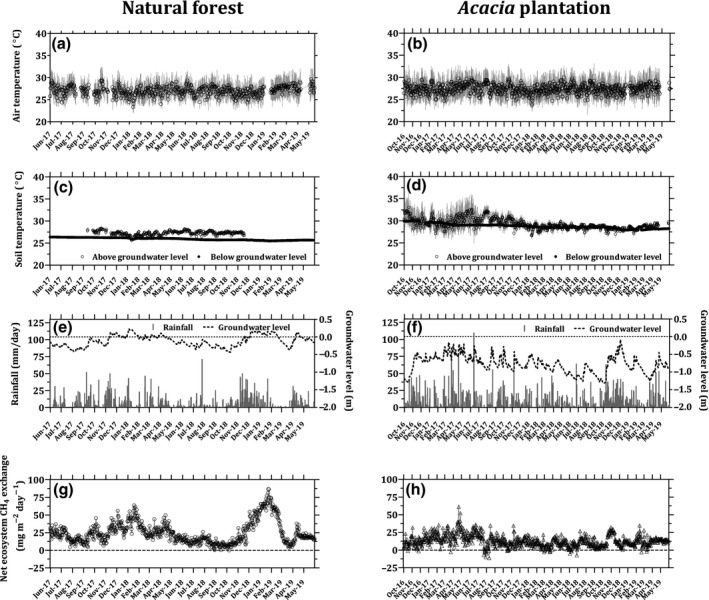
Variations in daily air temperature (a, b), soil temperature above and below groundwater level (c, d), cumulative rainfall and groundwater level (e, f), and net ecosystem CH_4_ exchanges (g, h) at the natural forest (left panels) and the *Acacia* plantation (right panels). The vertical bar in panels (a, b, c, d) represents standard deviation. Positive value of groundwater level indicates water level above the peat surface, and negative values indicate water level below the soil surface

Daily average *T*_air_ fluctuated between 23.3 and 29.9°C as a function of rainfall and cloud cover, without showing any clear seasonality (Figure 3a,b). Daily average *T*_soil_ above the GWL at the natural forest ranged from 25.6 to 28.3°C depending on the GWL and cloudiness, again without clear seasonality. At the *Acacia* plantation, daily average *T*_soil_ above the GWL ranged from 26.6 to 33.0°C as a function of canopy development, GWL, and cloudiness, without any clear seasonality (Figure 3c,d). The daily average *T*_soil_ above and below the GWL at the natural forest was statistically different (*t* test; *p* < .05; Table 3) and around ~2°C lower than at the *Acacia* plantation (Figure 3c,d). The average VPD at the natural forest of 3.7 ± 1.9 hPa was significantly lower (40%) than the average of 5.6 ± 2.2 hPa at the *Acacia* plantation (*t* test; *p* < .05; Table 3).

**Table 3:** The average and standard deviation of environmental variables

Site	Natural forest			*Acacia* plantation		
	Year 1 (June 2017–May 2018)	Year 2 (June 2018–May 2019)	Study period (June 2017–May 2019)	Year 1 (June 2017–May 2018)	Year 2 (June 2018–May 2019)	Study period (October 2016–May 2019)
Air temperature (°C)	26.9 ± 1.05	27.1 ± 0.9	27.0 ± 1.0	27.4 ± 1.1	27.4 ± 0.9	27.4 ± 1.0
Photosynthetic photon flux density (µmol m^−2^ s^−1^)	342 ± 90	328 ± 83	335 ± 87	343 ± 78	365 ± 83	358 ± 86
Vapor pressure deficit (hPa)	3.79 ± 2.23	3.72 ± 1.7	3.75 ± 1.98	5.85 ± 2.15	4.97 ± 2.31	5.52 ± 2.33
Canopy conductance to water vapor (mol m^−2^ s^−1^)	0.32 ± 0.11	0.34 ± 0.11	0.33 ± 0.11	0.29 ± 0.09	0.38 ± 0.1	0.32 ± 0.11
Cumulative rainfall (mm)	2,019	1,756	3,775	1,907	2,034	5,705
Groundwater level (m)	0.20 ± 0.13	0.28 ± 0.15	0.24 ± 0.14	0.69 ± 0.09	0.75 ± 0.17	0.73 ± 0.14
Soil temperature above groundwater level (at 15 cm below ground surface; °C)	27.2 ± 0.5	27.4 ± 0.3	27.3 ± 0.4	29.6 ± 1.3	28.5 ± 0.4	29.3 ± 1.2
Soil temperature below groundwater level (at 150 cm below ground surface; °C)	26.2 ± 0.1	25.7 ± 0.1	25.9 ± 0.3	28.8 ± 0.2	28.3 ± 0.3	28.8 ± 0.5
Wind speed (m/s)	2.12 ± 0.45	2.11 ± 0.43	2.11 ± 0.44	2.32 ± 0.56	1.98 ± 0.38	2.16 ± 0.53
Friction velocity (m/s)	0.2 ± 0.04	0.2 ± 0.04	0.2 ± 0.04	0.19 ± 0.04	0.17 ± 0.06	0.18 ± 0.05

At both sites, the daily cumulative rainfall was highly variable, ranging from 0 to 137 mm (Figure 3e,f), but did not significantly differ between sites (Mann–Whitney test; *p* > .05). Annual average rainfall was 1,887 and 1,970 mm for the natural forest and the *Acacia* plantation, respectively, similar to the previously reported average annual rainfall of the study area as a whole. GWL at the natural forest showed periodic sharp rises and steady decreases corresponding to rain events (Figure 3e). At the natural forest, the GWL rose up to 23 cm above the peat surface in the wet season, then in the late dry season reached −44 cm. The annual GWL pattern at the natural forest was almost the same as reported for other undrained peatland in Southeast Asia (Cobb et al., 2017). At the *Acacia* plantation, GWL rose up during rain events, but remained always below the ground surface (Figure 3f). During the study period, the average GWL from six sampling points around the natural forest tower of −0.24 ± 0.14 m was significantly shallower than that of −0.73 ± 0.14 m from 10–20 sampling points around the *Acacia* plantation tower (Mann–Whitney test; *p* < .05).

### 3.2 Net ecosystem CH_4_ exchanges

**Figure 4:**
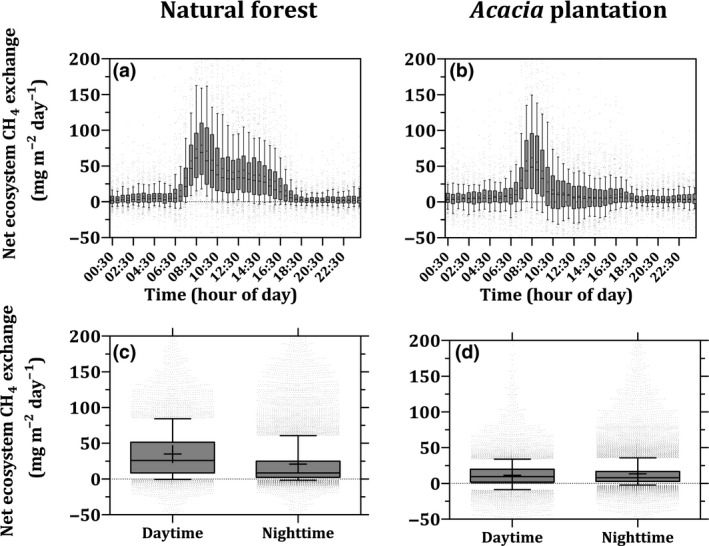
Diurnal variation in the net ecosystem CH_4_ exchanges (a, b), and daytime (10:30–18:30 hr) and nighttime (18:30–10:30 hr) ranges for net ecosystem CH_4_ exchanges (c, d) at the natural forest (left panels), and the *Acacia* plantation (right panels). The boxes show the median and the interquartile range, and whiskers denote the 10–90 range of all values

At both sites, the NEE‐CH_4_ showed a marked peak at around 07:00–10:30 hr (Figure 4a,b), consistent with flushing of CH_4_ accumulated in the vegetation canopy at night following the onset of turbulent mixing in the morning (Wong et al., 2018). NEE‐CH_4_ over the natural forest remained much higher than the nighttime during the remaining day hours and began to decline late in the afternoon (Figure 4a). NEE‐CH_4_ over the *Acacia* plantation began to decline and reached levels similar to the nighttime values after around 10:30 hr (Figure 4b). Thus, the diurnal variation in NEE‐CH_4_ was more pronounced over the natural forest (Figure 4a,b).

**Figure 5:**
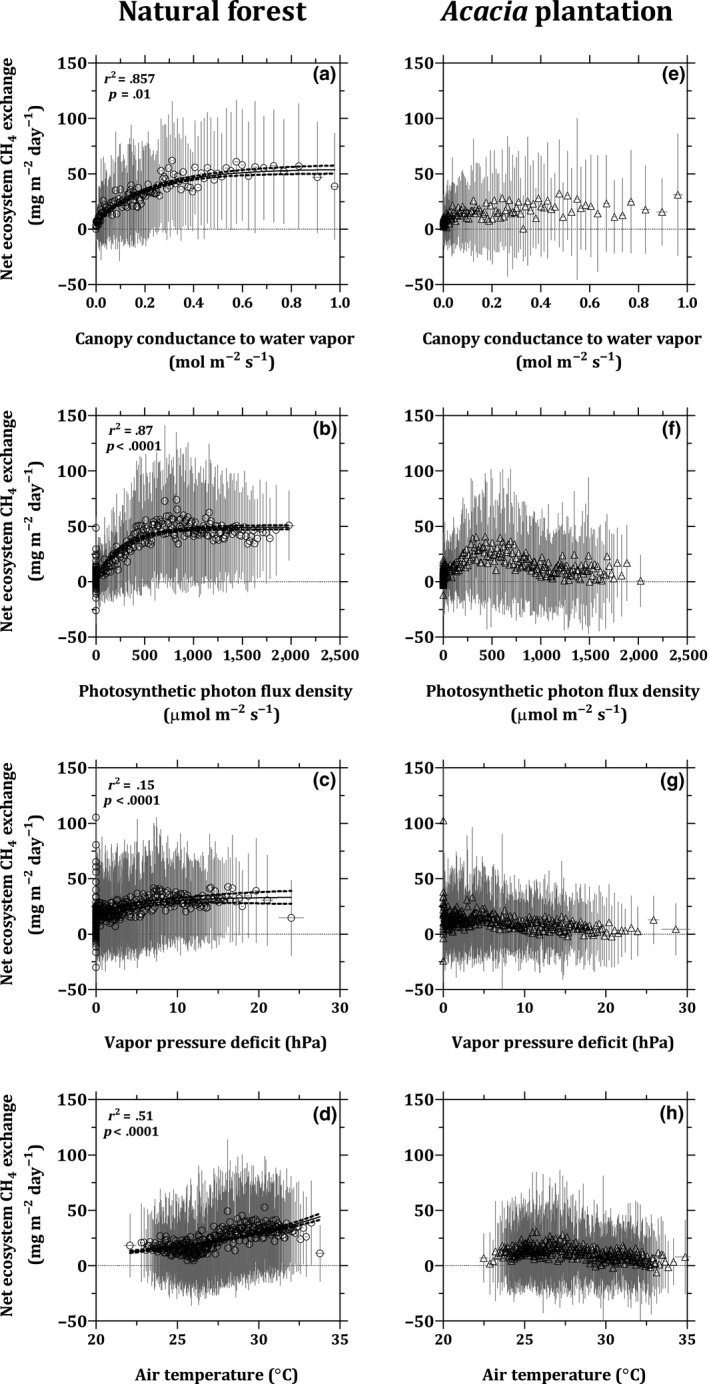
Response of the half‐hourly net ecosystem CH_4_ exchanges to canopy conductance to water vapor (a, e), photosynthetic photon flux density (b, f), vapor pressure deficit (c, g), and air temperature (d, h) at the natural forest (left panels), and the *Acacia* plantation (right panels). Data were binned by subgroups of 50 values of independent variable and corresponding net ecosystem CH_4_ exchange rates and then averaged for the subgroup. The vertical and horizontal bars represent the standard deviation for the subgroup. Note: we excluded measurements from 7:00 to 10:30 hr to avoid the possible bias due to flushing of nighttime accumulated CH_4_. The exclusion of data may have created biases in actual response curves of both ecosystems, but this bias would not change the interpretation

In order to avoid bias due to flushing of accumulated CH_4_, we considered nighttime NEE‐CH_4_ from 18:30 to 10:30 hr and daytime from 10:30 to 18:30 hr. This threshold might principally be site specific, but offered an opportunity to examine the diurnal variation in the NEE‐CH_4_ over our sites. Over the natural forest, daytime median NEE‐CH_4_ was more than three times higher (29 mg m^−2^ day^−1^) than at nighttime (8.4 mg m^−2^ day^−1^; Mann–Whitney test; *p* < .05; Figure 4c). Furthermore, daytime median NEE‐CH_4_ was almost three times higher over the natural forest than over the *Acacia* plantation (Mann–Whitney test; *p* < .05; Figure 4c,d). In contrast, the nighttime medians NEE‐CH_4_ were 8.3 and 7.9 mg m^−2^ day^−1^, respectively, over the natural forest and the *Acacia* plantation (Figure 4c,d). The diurnal variation in NEE‐CH_4_ over the natural forest was positively correlated with corresponding changes in canopy conductance to water vapor, PPFD, VPD, and *T*_air_ (Figure 5a–d). However, we did not observe a corresponding dependency of diurnal NEE‐CH_4_ on any environmental variables at the *Acacia* plantation (Figure 5e–h).

**Figure 6:**
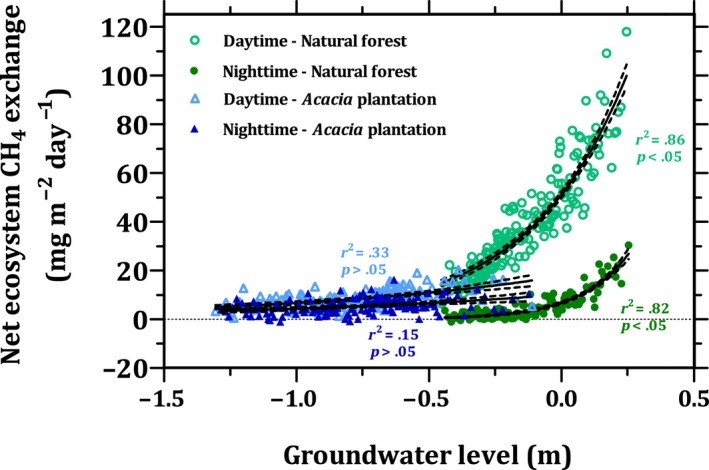
The relationship between the half‐hourly net ecosystem CH_4_ exchange and the groundwater level. Data were binned by subgroups of 50 values of groundwater level and corresponding net ecosystem CH_4_ exchange rates and then averaged for the subgroup. Note: we excluded measurements from 7:00 to 10:30 hr to avoid the possible bias due to flushing of nighttime accumulated CH_4_. The exclusion of data may have created biases in actual response curves of both ecosystems, but this bias would not change the interpretation

During the study period, daily NEE‐CH_4_ ranged from −0.15 to 86.6 mg m^−2^ day^−1^ and −11.3 to 61.2 mg m^−2^ day^−1^ at the natural forest and the *Acacia* plantation, respectively (Figure 3g,h). Daily NEE‐CH_4_ was almost two times higher (median = 20.7 mg m^−2^ day^−1^ and mean = 25.0 mg m^−2^ day^−1^) over the natural forest than over the *Acacia* plantation (median = 11.6 mg m^−2^ day^−1^ and mean = 12.8 mg m^−2^ day^−1^; Mann–Whitney test; *p* < .05; Table 4). Variation in daytime and nighttime NEE‐CH_4_ was positively correlated with associated changes in GWL at both sites (Figure 6). Notably, the relationships between NEE‐CH_4_ and GWL for daytime and nighttime over the natural forest were significantly different, whereas the relationships were quite similar over the *Acacia* plantation (Figure 6). There was no clear relationship between NEE‐CH_4_ and the *T*_soil_ either above or below the GWL (data not shown).

**Table 4:** Net ecosystem CH_4_ exchanges over the natural forest and the *Acacia* plantation using the eddy covariance technique. Daily average values are with standard error, whereas cumulative annual values are with cumulative flux uncertainty

	Gap‐filling approach	Natural forest			*Acacia* plantation			
		Year 1 (June 2017–May 2018)	Year 2 (June 2018–May 2019)	Average (June 2017–May 2019)	Year 1 (June 2017–May 2018)	Year 2 (June 2018–May 2019)	Average (June 2017–May 2019)	Study period including matured *Acacia* plantation and harvesting activity (October 2016–May 2019)
Daily average (mg m^−2^ day^−1^)	Mean diurnal course	26.0 ± 0.3	24.7 ± 0.3	25.3 ± 0.2	12.3 ± 0.2	12.0 ± 0.2	12.2 ± 0.1	13.6 ± 0.1
	Marginal distribution sampling	25.4 ± 0.2	23.7 ± 0.3	24.6 ± 0.2	10.4 ± 0.2	10.8 ± 0.1	10.6 ± 0.1	12 ± 0.1
	Average	25.8 ± 0.2	24.2 ± 0.3	25.0 ± 0.3	11.4 ± 0.2	11.4 ± 0.2	11.4 ± 0.1	12.8 ± 0.1
Cumulative annual (mg m^−2^ year^−1^)	Mean diurnal course	9,510 ± 637	8,997 ± 635	9,255 ± 899	4,476 ± 654	4,332 ± 917	4,404 ± 1,126	4,928 ± 1,444
	Marginal distribution sampling	9,280 ± 716	8,655 ± 615	8,967 ± 944	3,798 ± 693	3,938 ± 870	3,868 ± 1,112	4,547 ± 1,495
	Average	9,395 ± 677	8,826 ± 625	9,111 ± 922	4,137 ± 674	4,135 ± 894	4,136 ± 1,120	4,738 ± 1,470
Impact of *Acacia* plantation on CH_4_ emissions (mg m^−2^ year^−1^)	Average						−4,975 ± 1,451	−4,373 ± 1,735

Our measurements showed that the natural forest emitted 9.1 ± 0.9 g m^−2^ year^−1^ to the atmosphere (Table 4). Annual NEE‐CH_4_ over the *Acacia* plantation were approximately 50% lower than the natural forest, at 4.7 ± 1.5 g m^−2^ year^−1^, suggesting a net reduction of CH_4_ exchanges from natural forest to *Acacia* plantation of −4.4 ± 1.7 g m^−2^ year^−1^ (Table 4).

## 4 DISCUSSION

### 4.1 High GWL supports diurnal variability in NEE‐CH_4_

Our results show substantial and apparent diurnal variation in the NEE‐CH_4_ over the natural forest where GWL are high, highlighting the importance of high‐frequency ecosystem‐scale flux measurements. There is increasing evidence that woody vegetation can emit a significant CH_4_ amount to the atmosphere (Barba et al., 2018; Covey & Megonigal, 2019; Pangala et al., 2013, 2017; Pitz & Megonigal, 2017; Rice et al., 2010; Wang et al., 2016; Welch, Gauci, & Sayer, 2019). In tropical peatlands, the majority of root biomass occurs within the upper 50 cm of the peat column (Brady, 1997; Sulistiyanto, 2004), and dissolved CH_4_ in the root zone can be significant (100–1,500 µmol/L; Hoyt, 2017; Pangala et al., 2013). The magnitude of vegetation‐mediated transport seems to be directly regulated by a well‐connected root–stem pathway for the CH_4_ transport, although it is strongly (if not primarily) controlled by the availability of dissolved CH_4_ in the root zone (Covey & Megonigal, 2019; Pangala et al., 2013, 2017; Waddington, Roulet, & Swanson, 1996). At the natural forest site, *S. uliginosa*, *C. ferrugineum*, and *Syzygium* spp. are the dominant species; together they represent around 75% of the tall‐canopy vegetation. *Shorea uliginosa*, *Mesua* sp. 1, and *Xylopia fusca* emit significant CH_4_ in tropical peatlands (Pangala et al., 2013). *Mesua* sp. and *C. ferrugineum* belong to the same family. Thus, *S. uliginosa* and *C. ferrugineum* may have contributed significantly to the vegetation‐mediated transport.

When the root zone is inundated, changes in biological processes in vegetation driven by solar energy input might be the most important factors controlling diurnal variation in measured NEE‐CH_4_ (Figure 5a–d), as reported in northern peatlands (Chanton, Whiting, Happell, & Gerard, 1993; Garnet, Megonigal, Litchfield, & Taylor, 2005; Kim et al., 1998; Long et al., 2010; Whiting & Chanton, 1996) and recently reported over tropical peatland (Tang et al., 2018) and flooded forest (Dalmagro et al., 2019). At the natural forest, the observed positive correlation between NEE‐CH_4_ and canopy conductance to water vapor suggests that CH_4_ could be dissolved in the water, absorbed by the roots, transported with sap flow, and emitted through the stem by effervescence (Garnet et al., 2005; Nisbet et al., 2009). In addition, the positive correlation between NEE‐CH_4_ and PPFD, VPD, and temperature (Figure 5b–d) may suggest vegetation‐mediated transport through either diffusion or convective throughflow (Brix, Sorrell, & Orr, 1992; Chanton, Martens, Kelley, Crill, & Showers, 1992; Dacey, 1981). Our results are in line with a study in a temperate forested wetland which showed a sudden decrease in CH_4_ emissions from *Betula pubescens* after leaf loss, suggesting physiological control on gas transport (Pangala, Hornibrook, Gowing, & Gauci, 2015). Furthermore, labile organic compounds released from root tissues during photosynthesis and respiration can then be used as substrates by methanogenic archaea, contributing to the diurnal variation in NEE‐CH_4_ (Chanton et al., 1995; Christensen et al., 2003). Our study did not aim to conduct direct measurements to establish the relative importance of these different processes. Quantifying the pathway‐specific emissions and improving our understanding on the impact of root distribution by depth and dissolved CH_4_ concentration profile are important future study (Barba et al., 2018; Megonigal, Brewer, & Knee, 2019).

Nighttime and daytime NEE‐CH_4_ was positively correlated with associated changes in GWL at both sites (Figure 6). Nighttime NEE‐CH_4_ can be considered as the emissions from soil and water surfaces since there would be negligible vegetation‐mediated transport. A higher GWL may support larger CH_4_ concentration gradients between the peat surface and the atmosphere. Thus, GWL seems to be the key indirect control on CH_4_ emissions via diffusion from soil surfaces (Winton, Flanagan, & Richardson, 2017). Overall, the lack of a difference between nighttime NEE‐CH_4_ over the natural forest and the *Acacia* plantation can be attributed to the high GWL at the natural forest and to the potential presence of emissions from the water surfaces of ditches and canals in the *Acacia* plantation (Jauhiainen & Silvennoinen, 2012; Manning, Kho, Hill, Cornulier, & Teh, 2019). In addition, the higher soil temperature at the *Acacia* plantation might have increased CH_4_ production (Sjögersten et al., 2018), while the higher peat bulk density and the absence of a hollow‐hummock microtopography at the *Acacia* plantation might have lowered CH_4_ oxidation by increasing soil moisture content and lowering oxygen diffusion in the peat (Estop‐Aragones, Knorr, & Blodau, 2012). For these reasons, the *Acacia* plantation seems to produce higher nighttime CH_4_ emissions compared to the natural forest if the same range of GWL (−0.4 to −0.1) at both sites is considered. The difference between daytime and nighttime NEE‐CH_4_ can be attributed to vegetation‐mediated transport. Thus, our estimated vegetation‐mediated transport over the natural forest is 71% of the total daytime emissions, which is in line with the published range for tropical peatlands (Pangala et al., 2013). Overall, at the same GWL range, the natural forest emits higher CH_4_ as compared to the *Acacia* plantation during the daytime, most likely due to the presence of CH_4_ emitting trees (i.e., *S. uliginosa* and *C. ferrugineum*). However, it should be noted that only a few measurements are available for the *Acacia* plantation for the same range of GWL (Figure 6).

The influence of vegetation on CH_4_ emissions is strongly dependent on the GWL, and therefore, the interaction among hydrology, vegetation, and CH_4_ emissions must be carefully taken into account for process‐based modeling (Figure 6). Predicted changes in rainfall amount, intensity, duration, and frequency and water management practices could affect the dynamics of hydrology in tropical peatlands (Ge et al., 2019), and thereby CH_4_ emissions (Saunois et al., 2016).

### 4.2 GWL controls seasonal variability in NEE‐CH_4_

The seasonal variation is controlled by the GWL driven by rainfall. Our results show higher NEE‐CH_4_ during the wet season as compared to the dry season. Other eddy covariance studies in tropical peatlands have reported a similar seasonal pattern in CH_4_ emissions (Sakabe et al., 2018; Wong et al., 2018). A study in Amazonian peatland reported lower soil‐CH_4_ emissions in the wet season as compared to the dry season, where the GWL was 54 cm above the peat surface during the wet season (Teh, Murphy, Berrio, Boom, & Page, 2017). If GWL rises above a limit, soil CH_4_ emissions can decrease with flooding depth as gas diffusion may be restricted more as hydrostatic pressure increases along with increasing flooding depth (Ishikura et al., 2019). Furthermore, the standing water can enhance CH_4_ oxidation because it would increase dissolved oxygen and prolong traveling time of CH_4_ to the atmosphere (Strack, Waddington, & Tuittila, 2004). Notably, Teh et al. (2017) only reported emissions from soil and water surfaces and did not measure vegetation‐mediated transport which can be significant in Amazonian wetlands (Pangala et al., 2017). This highlights that seasonality differs from one pathway to another; thus, caution should be taken when modeling seasonality in CH_4_ emissions from tropical peatlands.

In northern peatlands, temperature exerts a strong effect on seasonal variation in CH_4_ emissions with an exponential dependence via its influence over enzyme kinetics of CH_4_ production and plant growth and development (Desai et al., 2015; Rinne et al., 2007; Tagesson et al., 2012). Observed fluctuations in both *T*_air_ and *T*_soil_ in this study are much smaller than those of northern peatlands. During the study period, the *T*_soil_ below the GWL varied within a very narrow range (~2°C) at both sites. This suggests that variation in *T*_soil_ would have only a minor effect (if any) on variation in NEE‐CH_4_. Furthermore, *T*_soil_ tended to be higher when GWLs were lower; thus, it is difficult to determine the independent effect (if any) that a change in temperature had on CH_4_ production and oxidation (Sjögersten et al., 2018). For example, if CH_4_ oxidation above the GWL increased more rapidly (due to the combination of a deeper aerobic zone and higher rates of microbial activity at a higher temperature) than rates of CH_4_ production below the GWL, the net effect of warmer and drier conditions would be a lower NEE‐CH_4_. Our results suggest that the effects of changing rainfall and land management on peat hydrology will be more important than rising temperature as a driver of changes in tropical peatland CH_4_ balance in the future.

### 4.3 Low GWL reduces NEE‐CH_4_ over the *Acacia* plantation

At the *Acacia* plantation, the lower GWL leads to an aerobic root zone (indeed, this is the specific aim of water management in the plantation, to support *Acacia* growth) which is likely to reduce (but not eliminate) CH_4_ production and transport. Firstly, aerobic conditions are unfavorable to methanogens and promote methanotrophy (Furukawa et al., 2005; Moore & Roulet, 1993; Strack et al., 2004). Secondly, as most of *Acacia* roots are mainly restricted above GWL in the aerated peat layer, this may result in inadequate CH_4_ in the root zone to be taken and transported to the atmosphere. But given the GWL fluctuation, it is possible that when GWL rises after a heavy rain event, some portion of the root system will be below GWL, at least for a few days. However, our measurements over the *Acacia* plantation do not show a diurnal variation in NEE‐CH_4_, and this may confirm that the root system remained above the GWL. Finally, it is likely that a substantial fraction of CH_4_ emission from the *Acacia* plantation area could be occurring from the open water surface of the ditch and canal network (Evans, Renou‐Wilson, & Strack, 2016; Jauhiainen & Silvennoinen, 2012; Manning et al., 2019), and therefore subject to different environmental controls (Deshmukh et al., 2014). The CH_4_ uptake rates in the *Acacia* plantation are similar to those previously reported over tropical peatlands during the dry season (Sakabe et al., 2018). The CH_4_ uptakes might be due to methanotrophy in the aerobic upper peat layer (Arai et al., 2014). However, CH_4_ uptake by tree may also be possible (Sundqvist, Crill, Mölder, Vestin, & Lindroth, 2012).

### 4.4 Potential effects of GWL on CH_4_ production and oxidation

Variation in soil redox conditions driven by GWL fluctuation plays an essential role in influencing not only the quantity but also the quality of organic substrate used by the methanogenic archaea for CH_4_ production (Girkin et al., 2018; Hoyos‐Santillan et al., 2016; Reiche, Gleixner, & Küsel, 2010; Winton et al., 2017). Higher GWLs promote CH_4_ production in a relatively large portion of the peat column and restrict the zone in which aerobic CH_4_ oxidation can occur (Moore & Roulet, 1993; Moore et al., 2011; Strack et al., 2004). In contrast, lower GWL would narrow the zone of CH_4_ production in the peat column and further supporting aerobic CH_4_ oxidation above the GWL. In tropical peatlands, the availability of labile organic matter is largely limited to near‐surface peat, for example, via root exudation and leaching from fresh litter (Brady, 1997; Könönen et al., 2016). When GWLs are low, most of this labile organic matter will be aerobically decomposed to CO_2_ (Itoh, Okimoto, Hirano, & Kusin, 2017) and unavailable for CH_4_ production. Therefore, when GWLs are low only organic matter with a greater aromatic content derived from the deeper peat would be available for anaerobic decomposition, restricting CH_4_ production (Sakabe et al., 2018). In the *Acacia* plantation, most of the labile organic matter supplied from harvested vegetation residues (leaf litter, small branches, and roots) and boiler wood ash might be restricted above GWL in the surface peat layer and expected to be aerobically decomposed to CO_2_ (Jauhiainen, Hooijer, & Page, 2012). Therefore, the effect (if any) of harvested vegetation residues and boiler wood ash on CH_4_ production would be minor.

### 4.5 Comparison of NEE‐CH_4_ with other studies

Our annual NEE‐CH_4_ over the natural forest are in the same range as those measured using the eddy covariance technique above a tropical peatland in the presence of CH_4_‐transporting trees (10.0–14.4 g m^−2^ year^−1^; Tang et al., 2018; Wong et al., 2018). In the absence of CH_4_‐transporting trees, a study in a tropical peatland reported no significant diurnal pattern in NEE‐CH_4_ (Sakabe et al., 2018) and far lower annual CH_4_ emissions (0.12–0.23 g m^−2^ year^−1^), despite similar GWLs to our forest site. The chamber‐based total ecosystem flux including tree CH_4_ emissions to an average height of 15 m based on the power function relationship from a tropical peatland is lower than our results over the natural forest (Pangala et al., 2013). Despite the higher GWL as compared to our study, the lower emissions in Pangala et al. (2013) can be attributed to (a) a lower hollow to hummock area ratio (1:1), as CH_4_ emissions from hollows can be up to 50 times higher as compared to hummocks (Pangala et al., 2013); and (b) possible underestimation of vegetation‐mediated transport. Emissions from young trees exceed those of mature trees by orders of magnitudes (Pangala, Gowing, Hornibrook, & Gauci, 2014), but Pangala et al. (2013) reported on emissions from mature trees. Also shoots can emit up to 10 times more than stems in a boreal forest (Machacova et al., 2016), but were not included in Pangala et al. (2013). Furthermore, entire trees may release CH_4_, albeit at the lower rates from their higher portions.

Our annual NEE‐CH_4_ over the natural forest are around two times higher than the IPCC CH_4_ emissions factor for rewetted tropical peatland, derived from undrained sites (Blain et al., 2014). This difference could be attributable to vegetation‐mediated transport, which was not captured by most of the studies used to derive the IPCC CH_4_ emission factor (Blain et al., 2014). Our annual NEE‐CH_4_ over the natural forest are nevertheless lower than those reported from Amazonian peatlands (Teh et al., 2017) and floodplain wetlands (Dalmagro et al., 2019; Pangala et al., 2017). In Amazonian peatlands, CH_4_ production is greater owing to high nutrient status and soil pH, and low recalcitrant carbon (Wassmann et al., 1992). In addition, methanotrophy is generally less effective because of increased anoxic and stratified, water‐submerged sediments (Bartlett et al., 1988; Devol & Rickey, 1990).

Our annual NEE‐CH_4_ over the natural forest is similar to emissions from northern bogs (average = 9.5 g m^−2^ year^−1^) and around two times lower than CH_4_ emissions from northern fens (average = 20.5 g m^−2^ year^−1^; Abdalla et al., 2016). Higher temperatures in tropical peatlands favor greater humification, selective removal of reactive labile carbohydrates, and accumulation of aromatic content leading to a highly recalcitrant residual peat (Brady, 1997; Hodgkins et al., 2018). This results in low substrate availability for CH_4_ production in the woody peat where there is a high aromatic lignin content (Miyajima, Wada, Hanba, & Vijarnsorn, 1997; Sakabe et al., 2018). In northern peatlands, peat is mainly derived from mosses, sedges, and herbs which contain a high carbohydrate and lower aromatic content (Hodgkins et al., 2018). This supports higher CH_4_ production in northern peatlands, despite lower temperatures (Sundh, Nilsson, Granberg, & Svensson, 1994; Updegraff, Pastor, Bridgham, & Johnston, 1995). Tropical peatlands also typically have higher vertical and lateral recharge rates, driven by higher hydraulic conductivity than northern peatlands (Evans et al., 2014), making them susceptible to rapid flushing of the dissolved CH_4_ after rainfall. This could limit CH_4_ accumulation in near‐surface porewaters, reducing the potential for diffusion, ebullition, and vegetation‐mediated transport, but could increase emissions via drainage waters. In contrast, lower vertical and lateral recharge rates in northern peatlands support the buildup of dissolved CH_4_ concentrations, and result in substantial ebullition and a high CH_4_ concentration near the surface soil causing high diffusive and vegetation‐mediated transport (Hoyt, 2017).

Our annual NEE‐CH_4_ at the *Acacia* plantation is around 18 times higher than the IPCC CH_4_ soil‐derived emission factor for this category, which is mostly based on soil CH_4_ flux measurements (Drösler et al., 2014). The IPCC methodology does, however, provide a separate emission factor for CH_4_ emissions from drainage canals in tropical peatlands, of 225 g/m^2^ ditch surface area year^−1^. Based on 2.1% of the flux footprint area occupied by canals and ditches at the *Acacia* plantation, the water surfaces are contributed to generate an area‐weighted emission of 4.74 g m^−2^ year^−1^, which is very similar to our ecosystem‐scale estimate over the *Acacia* plantation. The *Acacia* plantation in this study is situated within one of the largest tracts of *Acacia* plantation in Southeast Asia, managed by the same company in a similar way. The fraction of water surface in the majority of land uses involving drainage in tropical peatlands is approximately 2% (Drösler et al., 2014). Hence, our *Acacia* plantation with 2.1% of water surface can be considered as both representative and conservative in Southeast Asia. The results indicate that despite their small fractional area, higher emissions from water management ditches could be large enough to partly compensate for the reduced CH_4_ emissions by lower GWL on the remainder of the managed peatland area. Notably, this suggests some potential for targeted mitigation measures to reduce CH_4_ emissions, for example, by keeping the ditches clear and vegetation free (Jauhiainen & Silvennoinen, 2012; Waldron et al., 2019).

Regardless of land‐cover on tropical peatland, if GWL is lower than 20 cm below the ground surface, most studies indicate that the peat acts as a net sink for atmospheric CH_4_ (Couwenberg, Dommain, & Joosten, 2010; Hergoualc'h & Verchot, 2012; Ishikura et al., 2019). Our results show that even when GWL is lower than 20 cm below the peat surface, both ecosystems acted as a CH_4_ source. Hence, it is possible for the ecosystem as a whole to act as a net CH_4_ source to the atmosphere due to emissions from vegetation and water surfaces, despite the soil surface likely acting as a net CH_4_ sink (Melling et al., 2005). Lower frequency and/or below‐canopy measurements, for example, daytime chamber measurements of soil surface exchange, are likely to give highly erroneous estimates of CH_4_ emissions in both ecosystems. In the case of the *Acacia* plantation, it is likely that such measurements would suggest that the system is a net sink for CH_4_, when in fact it remains a source (albeit smaller than the natural forest).

Overall, our results highlight that tropical peatlands, including natural forest and areas managed for forest plantation, are significant sources of CH_4_, and probably have a greater impact on global atmospheric CH_4_ concentrations than previously thought. The associated radiative forcing effect of CH_4_ emissions has the potential to partly offset net CO_2_ uptake.

If we follow IPCC GWP accounting methodology and apply a 100 year GWP of 34 for CH_4_ (Myhre et al., 2013), this implies a CH_4_ emission of 3.1 t CO_2_eq ha^−1^ year^−1^ from natural forest. Applying a long‐term peat accumulated CO_2_ rate of around 2.6 t CO_2_ ha^−1^ year^−1^ since their formation (Dommain et al., 2011), the 100 year net warming impact for tropical peatland would be 0.5 t CO_2_eq ha^−1^ year^−1^. Over longer time‐horizons, the shorter atmospheric lifetime of CH_4_ compared to CO_2_ means that an ecosystem that is in approximate greenhouse gas balance based on 100 year net warming impact will have a net cooling impact if it acts as a sustained CO_2_ sink and a steady CH_4_ source (Allen et al., 2018; Frolking, Roulet, & Fuglestvedt, 2006). However, according to the current IPCC assessment, tropical peatlands are in approximate CO_2_ balance (Drösler et al., 2014); therefore, the net warming impact value would be 3.1 t CO_2_ ha^−1^ year^−1^. Nevertheless, our data confirm that CH_4_ emissions from tropical peatlands should be included in landscape level greenhouse gas budgets (Miettinen, Hooijer, Vernimmen, Liew, & Page, 2017; Wijedasa et al., 2018).

### 4.6 Impact of *Acacia* plantation on CH_4_ emissions

We present here an assessment of the impact of forest plantation on CH_4_ emissions associated with the altered landscape (i.e., *Acacia* plantation). By definition, the impact represents the actual CH_4_ exchange with the atmosphere in addition to the exchange that existed in the pre‐existing natural landscape, and thus represents the exchange that can be directly attributed to the creation and existence of the *Acacia* plantation (Prairie et al., 2018; Teodoru et al., 2012).

**Figure 7:**
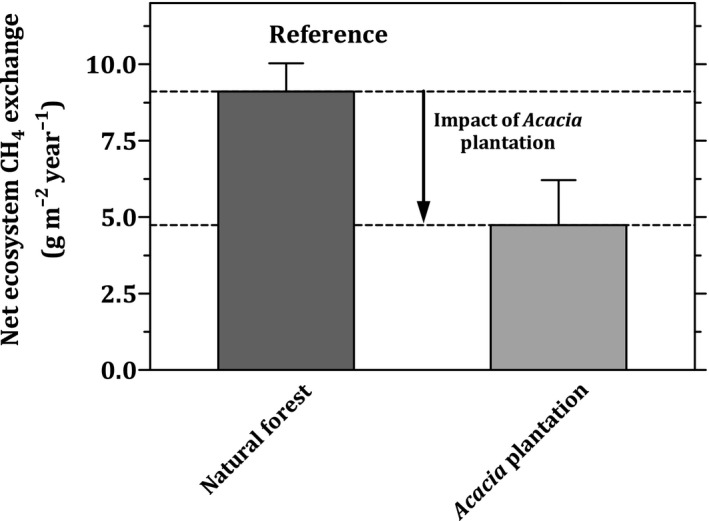
Impact of the *Acacia* plantation on net ecosystem CH_4_ exchange from tropical peatland

Our measurements indicate that both studied ecosystems in the tropical peatland functioned as net CH_4_ sources to the atmosphere (Table 4, Figure 7). Therefore, our results indicated that the impact of the *Acacia* plantation was to reduce CH_4_ emissions by 4.4 ± 1.7 g m^−2^ year^−1^ (Table 4, Figure 7). If we apply a 100 year GWP of 34 (Myhre et al., 2013), this implies an emission reduction of 1.5 t CO_2_eq ha^−1^ year^−1^. For comparison, the IPCC’s Tier 1 default emission factor for CO_2_ from *Acacia* plantation on tropical peat is 73 t CO_2_ ha^−1^ year^−1^ (Drösler et al., 2014), which is larger than the natural forest. Measurements of net ecosystem CO_2_ exchanges over the natural forest and the *Acacia* plantation are being conducted (C. Deshmukh, unpublished data); results of this ongoing study will be published in due course, following the completion of one 5 year *Acacia* plantation cycle, and will also take into consideration the biomass harvested from the plantation. These measurements will lead to a better understanding of the climate footprint of *Acacia* plantation (Dommain et al., 2018; Petrescu et al., 2015).

The estimated impact of *Acacia* plantation on CH_4_ exchange related to land‐cover change that we present here is by no means invariant in time and space. In addition to variations related to natural hydrology, the impact is also likely to vary with actual water management practices in plantation landscapes. Furthermore, results presented here are specific for *Acacia* plantation; thus, caution should be taken when extrapolating to other agriculture in the region (e.g., sago, oil palm, rubber plantations, etc.) with different water management practices and fertilizer applications (Hergoualc'h & Verchot, 2012). To evaluate the impact of land‐cover change on global peatland CH_4_ emissions, more ecosystem‐scale flux measurement studies are needed.

In conclusion, our half‐hourly multi‐year NEE‐CH_4_ measurements directly captured and integrated “hot spot and hot moment” dynamics of all known and unknown sources and removals in the studied ecosystems. The observed high variability in NEE‐CH_4_ suggests complex nonlinear process‐level controls on CH_4_ exchange between tropical peatlands and the atmosphere. Our results provide some of the first reliable information on the magnitudes of CH_4_ exchange at a tropical peatland ecosystem scale, demonstrating that traditional manual soil chamber techniques provide an incomplete picture of the total CH_4_ flux, and improving mechanistic understanding based on high temporal resolution measurements of NEE‐CH_4_ and key environmental variables such as the sensitivity of emissions to GWL. Our data indicate that the *Acacia* plantation on tropical peatland results in significant reductions in CH_4_ emissions compared to the natural system, although the associated cooling impact is likely to be smaller than the accompanying warming impact of higher CO_2_ and nitrous oxide emissions. More ecosystem‐scale measurements are needed to fully evaluate the effect of land‐cover change on the greenhouse gas balance, at a larger number of sites and over long time periods, in order to develop science‐based, climate‐smart management practices for tropical peatlands.

## Data Availability

The data that support the findings of this study are available from the corresponding author upon reasonable request.
